# Initial and Middle Stages of Quantum Dots Growth: From Dynamics of Superstructures to Island-Size Distributions

**DOI:** 10.3390/nano16090510

**Published:** 2026-04-23

**Authors:** Olzhas Kukenov, Vladimir Dirko, Kirill Lozovoy, Andrey Kokhanenko

**Affiliations:** Department of Quantum Electronics and Photonics, Faculty of Radiophysics, National Research Tomsk State University, Lenin Av. 36, 634050 Tomsk, Russia; okukenov@mail.ru (O.K.); vovenmir@gmail.com (V.D.); kokh@mail.tsu.ru (A.K.)

**Keywords:** quantum dots, 2D layers, molecular beam epitaxy, reflection high-energy electron diffraction, germanium, silicon

## Abstract

The dynamics of initial layer-by-layer growth and subsequent nucleation of quantum dots of Si and Ge on Si(001) were studied combining reflection high-energy electron diffraction, scanning electron microscopy and atomic force microscopy. It was shown that the processes occurring at the initial stage determine further growth of the heterostructure and final shape and density of nanoislands. The mechanisms of terrace formation, occurrence and dynamics of dimer rows of the 2 × *N* superstructure, and effects of temperature on the growth characteristics were described. The obtained experimental dependences show the critical relationship between the synthesis parameters (growth temperature), epitaxial growth processes and the characteristics of the resulting nanoislands. The fundamental studies conducted make it possible to create self-organizing quantum dots of a given size and density for advanced optoelectronics, including infrared photosensitive elements and single-photon detectors.

## 1. Introduction

The development of the semiconductor electronics industry is closely linked to the study of fundamental processes and phenomena. Envisioning the contemporary world without computers, gadgets, various detectors, and communication systems is hardly possible, as they have become an integral component of nearly all aspects of modern society. All of this has become possible thanks to advances in semiconductor electronics. Improving the properties of electronic components is a key task for the global scientific community today, as progress in many other fields requires ever-increasing computing power, specific absorption spectra, and so on [1]. Indeed, from medicine to art, electronic components are used in one way or another. Improving devices is possible not only by increasing the number of components or using specific and often expensive materials, but also by creating nanostructures [2].

The vast majority of electronic components are based on silicon due to its availability and resistance to external influences [3,4]. Germanium has high charge carrier mobility and a small difference (~4.2%) in lattice constant compared to Si, which makes Ge highly promising for integration into the silicon industry [5,6]. Using Ge and Si, manufacturing of various types of nanostructures, such as quantum wells, quantum wires, and quantum dots (QDs), is possible. Size quantization leads to new properties in these materials, which allows for the creation of more efficient devices based on them [7,8]. Germanium quantum dots on Si substrates find applications, for example, in solar energy, fiber-optic communication lines, and spintronic elements [9,10,11]. Advances in semiconductor nanoelectronics are inherently coupled with increasingly stringent demands on the structural quality and dimensional precision of fabricated nanostructures. Therefore, it is especially important to create quantum dots of a given size and with the required density. These parameters directly depend on the fundamental processes occurring during their synthesis.

Molecular beam epitaxy (MBE) remains the benchmark technique for fundamental studies of QDs growth due to its unparalleled control over growth kinetics, surface processes, and in situ monitoring. But it has limitations in terms of scalability, throughput, and industrial compatibility [12]. In contrast, metal–organic chemical vapor deposition (MOCVD) offers clear advantages in terms of industrial scalability, wafer uniformity, and compatibility with existing semiconductor fabrication lines [13,14]. The transition from MBE to MOCVD is not trivial and requires careful consideration of growth regimes. The key strategy here is not a direct replication of MBE growth conditions, but rather the identification of robust physical mechanisms (e.g., strain-driven nucleation, surface diffusion anisotropy, critical thickness behavior) that can be applied to MOCVD growth processes.

Reflection high-energy electron diffraction (RHEED) allows for the monitoring of surface morphology during growth with an accuracy better than one monolayer (ML) [15]. RHEED is used to analyze both a thin surface layer of the sample and the overall surface morphology. It is a universal surface monitoring method used by scientists worldwide [16,17,18,19,20].

The formation of Ge/Si QDs via the Stranski–Krastanow growth mode is inherently governed by stochastic nucleation processes driven by the interplay between strain relaxation, surface diffusion, and local fluctuations in adatom density. As demonstrated in seminal studies [21,22,23], nucleation occurs at randomly distributed sites once the critical wetting layer thickness is exceeded, leading to intrinsic dispersion in island size, shape, and spatial distribution. This stochasticity fundamentally limits the degree of lateral ordering and results in broad size distributions.

It is well established that surface reconstruction, step density, and defect distribution critically influence adatom migration and preferential nucleation sites. Approaches such as substrate patterning, vicinal surfaces, and pre-structured templates have been proposed to suppress stochastic nucleation and improve ordering. Similarly, modulation of growth parameters, including temperature, deposition rate, and surfactant use, has been shown to partially narrow the size distribution, although complete suppression of disorder remains challenging due to the thermodynamic nature of self-assembly. Numerous studies describe the basic processes and phenomena [24,25,26], but some new, unexpected results continue to be explored. For example, formation of ultrathin germanium nanowires [27], synthesis of ensembles of GeSi nanoislands with monomodal size distribution at high temperatures [28,29], preferential growth of quantum dots at the periphery of Si microdisks [30,31], and elastic interaction between quantum dots [32,33]. Controlling epitaxial processes using temperature, deposition rate, or substrates with a specific misorientation angle will allow the creation of self-organized quantum dots with the required parameters and desired properties.

Recently, particular attention has been paid to the development of photosensitive elements based on germanium-silicon materials, including avalanche photodiodes and single-photon detectors based on Ge/Si(001) with germanium quantum dots [34,35,36]. The properties of the resulting photodetectors depend on the parameters of the quantum dots and the surface state of the epitaxial films. In turn, epitaxial processes influence the quantum dot parameters. For example, monochromatic photodetectors require heterostructures with a narrow nanoisland size distribution. The homogeneity of Ge quantum dots on Si(001) is influenced by growth parameters, such as synthesis temperature, growth rate, the amount of deposited germanium, and post-growth annealing of the structure. The desired nanoisland size distribution can be achieved by carefully selecting and continuously monitoring growth conditions.

On the Si(001) surface, two distinct types of terraces alternate, differing in their reconstruction and dimer orientation. Type A terraces exhibit a 1 × 2 reconstruction, in which the dimer rows are aligned parallel to the step edge, whereas type B terraces are characterized by a 2 × 1 reconstruction with dimer rows oriented perpendicular to the step direction [37]. During homoepitaxial deposition of Si on Si(001), the addition of each atomic layer induces an alternation of terrace types, effectively converting a terrace into one of the opposite reconstruction. Under specific combinations of substrate temperature and deposition rate, step bunching may occur, leading to step doubling; as a consequence, terraces of one type can become markedly wider than those of the complementary type.

Although the growth of Ge on Si has been extensively investigated, several fundamental issues remain insufficiently understood, which is increasingly critical in light of stringent requirements for nanostructure quality. In particular, the mechanisms governing elastic strain relaxation in the Ge layer on Si are not yet fully clarified. The lattice mismatch between Ge and Si generates significant strain, which is partially relieved through morphological evolution during epitaxial growth.

At the early stages, strain relaxation proceeds via the formation of surface reconstructions such as 2 × *N* and *M* × *N* superstructures. Upon completion of the wetting layer, the growth mode changes, and a transition from two-dimensional layer-by-layer growth to three-dimensional island formation occurs [38]. However, questions regarding the length of the 2 × *N* superstructure at different temperatures during epitaxial growth of Ge on Si(001), as well as its relationship with the critical thickness of the transition from two-dimensional to three-dimensional growth still remain controversial.

Numerous studies of heteroepitaxial growth of germanium on silicon show that, under certain conditions, several morphologically different types of islands can form in this system: with a square and rectangular base [38]. Furthermore, under certain growth conditions, the formation of so-called quantum wires is possible, which have a very high length-to-base width ratio [39]. Growth temperature is key factor in all these processes.

This work aims to establish the relationship between synthesis parameters, growth processes, and quantum dot parameters. This includes determining the temperature dependences of the epitaxial growth characteristics of Si and Ge on Si(001) and determining the effect of temperature on the parameters of the synthesized quantum dots. Controlling the geometric factors and density of quantum dots will enable the creation of unique detectors for a wide range of applications.

The results of this study contribute to our understanding of the kinetics of superstructure transitions during the synthesis of heteroepitaxial germanium films on silicon, as well as the mechanisms of Si and Ge terrace formation on Si(001) using molecular beam epitaxy. These results provide the potential for developing a process for producing germanium- and silicon-based opto- and nanoelectronic devices using molecular beam epitaxy.

## 2. Materials and Methods

The synthesis was carried out in a Katun-100 ultrahigh-vacuum molecular beam epitaxy unit (ISP SB RAS, Novosibirsk, Russia) equipped with two electron beam evaporators for silicon (purity 7N) and germanium (6N). Commercially available Si(001) wafers (MTI Corporation, Richmond, CA, USA) with misorientation of less than 0.1° from the crystallographic plane were used as a substrate (Figure S1). Chemical cleaning of the silicon wafers involved removing the native oxide layer in a dilute HF (7N) solution, followed by the formation of a controlled oxide layer in an NH_4_OH(6N):H_2_O_2_(5N):H_2_O solution. At all stages, the wafers were rinsed with a stream of deionized water with a resistivity of at least 18 MΩ·cm. The chemically cleaned silicon wafers (Figure S2) were immediately placed in the epitaxy chamber for thermal annealing of the oxide layer.

Molecular beam epitaxy enables the production of clean, thin layers of a defined thickness with a minimal number of defects and sharp heterojunctions. The ultra-high vacuum conditions of MBE allow for the use of specialized control methods.

Reflection high-energy electron diffraction is one of the main analytical methods used in this work due to its versatility and compatibility with molecular beam epitaxy. The principle of the method is based on directing a beam of high-energy electrons at a small grazing angle onto the surface. The wavelength of such electrons is commensurate with the parameters of the crystal lattice. Therefore, upon reflection from the sample, the electrons form a diffraction pattern on a luminescent screen. The nature of this diffraction pattern can be used to infer the surface morphology [40,41,42]. RHEED is applicable to the analysis of superstructural changes, determination of deposition rates and growth mechanisms, investigation of changes in lattice parameters, estimation of composition of solid solutions, determination of the critical thickness of epitaxial layers, and analysis of the faceting of formed quantum dots. In this work, RHEED was used to analyze the growth of thin two-dimensional layers and quantum dots during epitaxy.

Scanning electron microscopy (SEM) and atomic force microscopy (AFM) are methods that allow high-resolution imaging of sample surfaces. In this study, a scanning electron microscope Apreo S LoVac (Thermo Fisher Scientific, BRNO, Czech Republic) and an atomic force microscope with a vacuum chamber Solver HV (NT-MDT, Moscow, Russia) were used to analyze quantum dots after their synthesis. No metal coating was applied prior to SEM imaging. Super sharp NSG30_SS (TipsNano, Moscow, Russia) cantilevers with tip’s curvature radius of 2 nm were used for atomic-force microscopy in tapping mode. Linear background subtraction was applied to 2D AFM images. For 3D AFM views smoothing, filter based on convolution with a Gaussian function was used. Gwyddion (v. 2.70) and ImageJ (v. 1.54g) software were used for microscopy images processing.

Due to the inevitable presence of defects on the silicon surface, a buffer layer thicker than 50 nm is required to obtain a smooth and defect-free surface. Pre-epitaxial surface preparation, including wafer cleaning and buffer layer deposition, effectively reduces the defect density associated with impurities and dislocations. Nevertheless, the formation of atomic steps on the surface cannot be avoided under these conditions [43,44,45,46]. These surface steps arise from the misorientation of the wafer surface relative to the Si(001) crystallographic plane. Since the surface is not perfectly smooth, these steps contribute to atomic kinetics during growth.

## 3. Results and Discussion

### 3.1. RHEED Analysis of Homoepitaxial Si/Si(001) Growth

Homoepitaxial deposition of Si on the Si(001) surface leads to the formation of alternating terraces of two types, denoted *T_A_* and *T_B_*, which are characterized by 1 × 2 and 2 × 1 surface reconstructions, respectively. These terraces differ in the orientation of dimer rows, which are aligned either parallel or perpendicular to the terrace edge [47,48]. As growth proceeds, the incorporation of each successive atomic layer induces a reversal of the terrace type, such that a given terrace transforms into one with the opposite reconstruction. In order to determine the surface morphology (i.e., the ratio of the widths of the two types of terraces) at different temperatures, experiments on Si growth on Si(001) were performed. Growth was carried out at a rate of 0.09 ML/s and temperatures ranging from 200 °C to 800 °C.

**Figure 1:**
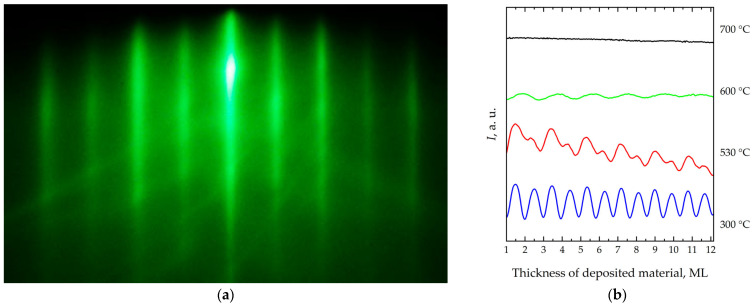
(**a**) Diffraction pattern from Si(001) surface in the [110] electron beam direction. (**b**) Diffraction intensity oscillations *I* near the specular reflection 00 as a function of the thickness of deposited material during the homoepitaxial Si/Si(001) growth in the [110] azimuth for various growth temperatures: 300 °C, 530 °C, 600 °C, and 700 °C.

RHEED analysis of the surface during homoepitaxial Si/Si(001) growth in the [110] azimuth shows a transition to a bimodal character of diffraction intensity oscillations at certain temperatures (Figure 1).

At low temperatures, regular oscillations corresponding to the growth of one monolayer of deposited material are observed. At intermediate temperatures, two types of maxima with different intensities and oscillation periods are observed. The higher maximum corresponds to the formation of the type B terraces with a 2 × 1 superstructure and dimer rows oriented perpendicular to the step edge, whereas the lower maximum corresponds to the formation of the type A terraces with a 1 × 2 superstructure and dimer rows parallel to the step edge [49,50,51]. At temperatures of about 550 °C, the lower maxima disappear. Finally, at temperatures higher than 700 °C, oscillations are no longer observed.

**Figure 2:**
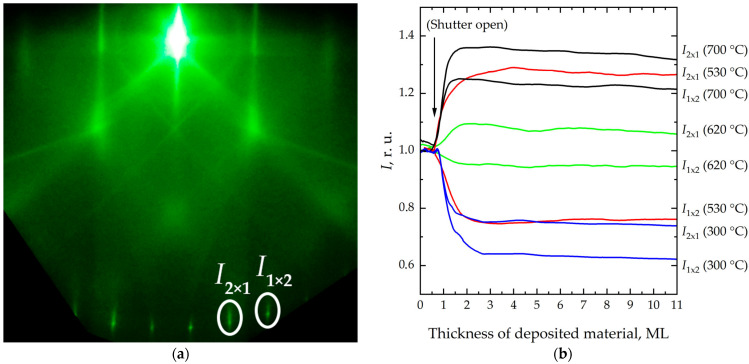
(**a**) Diffraction pattern from Si(001) surface in the direction of the electron beam [100]. (**b**) Change in the intensity *I* of reflections from 1 × 2 and 2 × 1 superstructures as a function of the thickness of deposited material during the homoepitaxial Si/Si(001) growth in the [100] azimuth for different growth temperatures: 300 °C, 530 °C, 620 °C, and 700 °C.

Next, the time dependences of the reflection intensities *I* in the [100] direction from the 2 × 1 and 1 × 2 superstructures during Si/Si(001) epitaxy were obtained over a wide temperature range (Figure 2). These dependences are consistent with previously reported data [46,49].

**Figure 3:**
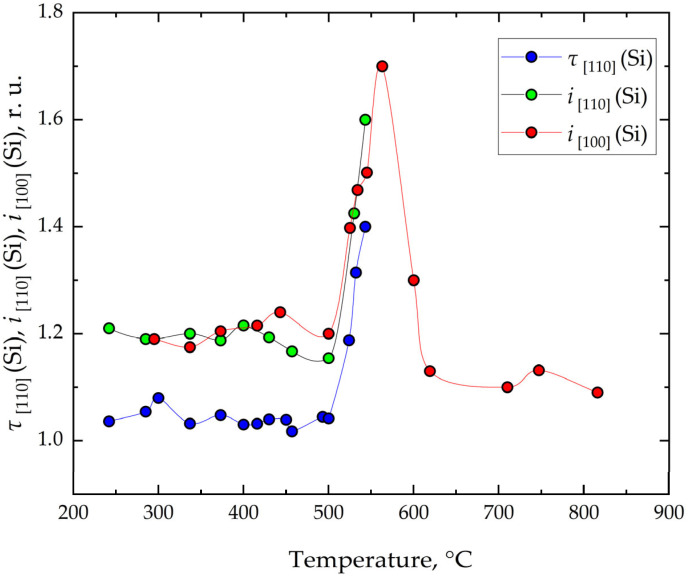
Dependences of the ratios of periods τ_[110]_ and intensities of oscillations *i*_[110]_ in the [110] azimuth and the ratios of intensities of reflexes from 2 × 1 and 1 × 2 superstructures *i*_[100]_ in the [100] azimuth on growth temperature.

In the RHEED study of the homoepitaxial Si/Si(001) growth in the direction of electron beam [110], the dependences of the intensity ratio of local maxima from terraces *T_B_* and *T_A_* (*i*_[110]_ = *I_B_*/*I_A_*) and the ratio of oscillation periods for the growth of terraces *T_B_* and *T_A_* (τ_[110]_ = τ*_B_*/τ*_A_*) were determined at low and intermediate growth temperatures (up to 550 °C) (Figure 3, blue and green dots). The temperature dependence of the intensity ratio of the reflections from the 2 × 1 and 1 × 2 superstructures in the [100] azimuth (*i*_[100]_ = *I*_2×1_/*I*_1×2_) was obtained for Si epitaxy on Si(001) over a wide temperature range (Figure 3, red dots). All these dependences exhibit similar behavior with a pronounced maximum near 550 °C.

In the low-temperature range (200–500 °C), temporal intensity oscillations were analyzed along the [110] azimuth. The ratio of oscillation periods associated with the 2 × 1 and 1 × 2 reconstructions slightly exceeds unity, with an average value of ~1.05, indicating that the formation time of one type of step is approximately 5% longer than that of the other. Under a constant deposition rate (0.09 ML/s), this difference directly reflects the ratio of step areas. Since these areas are nearly equal, step bunching and convergence are negligible in this regime. As a result, step-flow growth is largely suppressed, and surface evolution is governed mainly by two-dimensional nucleation. Due to limited adatom diffusion lengths, many adatoms fail to reach step edges and instead aggregate into 2D islands, leading to increased surface roughness [52,53]. The oscillation amplitudes corresponding to different terrace types differ by a factor of ~1.22, which can be attributed not only to terrace width but also to variations in electron reflectivity. In contrast, along the [100] azimuth, the intensity ratio between 2 × 1 and 1 × 2 reflections (~1.21) remains insensitive to terrace reflectivity, as the dimers are oriented at 45° relative to the incident electron beam [53].

As the temperature increases to 500–560 °C, the ratio of oscillation periods increases, indicating the onset of step convergence. In this intermediate regime, growth proceeds via a combination of mechanisms: adatoms partially incorporate into step edges, while the remaining fraction contributes to the formation of two-dimensional islands. RHEED data along the [110] azimuth still indicate surface roughness, although it is less pronounced than at lower temperatures.

Within the 560–600 °C interval, the previously observed bimodal oscillation pattern in the [110] direction disappears and is replaced by oscillations with a doubled period. Simultaneously, the intensity contrast between the 2 × 1 and 1 × 2 reflections in the [100] azimuth decreases with increasing temperature. It is assumed that, as the temperature rises, adatoms reaching a step—due to their increased diffusion length—more frequently find kink sites for incorporation. If the diffusion length or kink density are insufficient, adatoms may either jump over a step or contribute to the formation of new kinks. Alternatively, in this temperature range, the adatom energy may become sufficient to overcome not only A-type steps but also B-type steps. As a result, steps that have approached each other may begin to separate as the temperature increases.

At higher temperatures (600–850 °C), oscillatory behavior is no longer observed, as enhanced adatom mobility ensures efficient incorporation at step edges. Under these conditions, the surface becomes smoother, and the intensities of the 1 × 2 and 2 × 1 reflections along the [100] azimuth become nearly indistinguishable.

Thus, the dominant step growth mechanism at a given temperature was identified. A critical temperature of approximately 550 °C was determined, at which monatomic steps of different types are closest to each other. This critical temperature weakly depends on the misorientation of the substrate and growth rate. Based on these findings, temperatures near 550 °C were selected for subsequent growth of Ge quantum dots on Si(001).

### 3.2. RHEED Analysis of Heteroepitaxial Ge/Si(001) Growth

**Figure 4:**
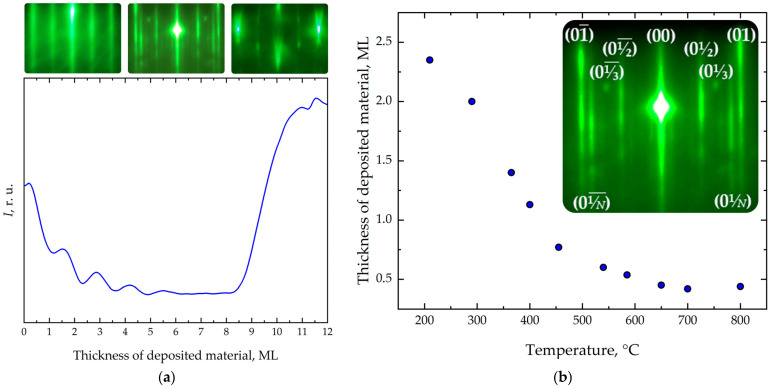
(**a**) Diffraction intensity *I* oscillations near the specular reflection 00 during the heteroepitaxial Ge/Si(001) growth in the [110] electron beam direction as a function of the thickness of deposited material. (**b**) Temperature dependence of the Ge coverage required for the appearance of the 1/*N* reflexes.

After evaluating the surface morphology during Si growth on Si(001) at different temperatures, we investigated the mechanisms of Ge growth on Si(001) at the same temperatures. The studies were conducted at a fixed misorientation angle of ~0.1° and a deposition rate of 0.02 ML/s. Since the lattice mismatch between these materials is approximately 4.2%, heteroepitaxial Ge/Si(001) growth proceeds via the Stranski–Krastanow mechanism. In this case, three-dimensional (3D) islands begin to nucleate after the formation of a two-dimensional (2D) wetting layer approximately 4 ML thick. During germanium deposition, the RHEED pattern in the [110] azimuth evolves with increasing coverage. Initially, the pattern reproduces that of the Si(001) surface, as the forming wetting layer replicates the substrate structure. Subsequently, 1/*N*-type reflexes appear (Figure 4a), indicating the formation of an ordered array of short dimer rows on the surface separated by dimer vacancies [48,54,55]. The transition from 2D to 3D growth is identified by an increase in intensity near the 01 reflection in the diffraction pattern (Figure 4a).

To assess the effect of temperature on the 2 × *N* superstructure, RHEED studies of the Si(001) surface in the [110] direction were performed during Ge deposition. It is known that the 1/*N* parameter requires a certain Ge layer thickness to reach a steady-state value [40]. In this work, we investigated the temperature dependence of the Ge coverage required for the appearance of the 1/*N* reflexes (Figure 4b) over the range 200–800 °C. The decrease in the values with increasing temperature (Figure 4b) is attributed to enhanced kinetic energy and surface mobility of adatoms, leading to an increased diffusion length. This facilitates the occupation of energetically favorable sites and promotes the relaxation of elastic strain arising from the lattice mismatch between Ge and Si.

**Figure 5:**
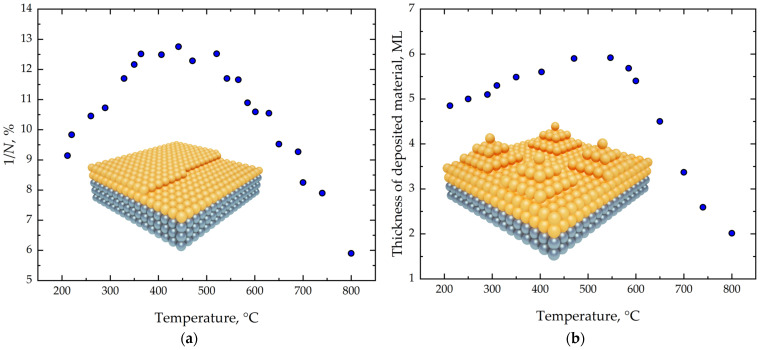
(**a**) Dependence of parameter 1/*N* value on growth temperature. (**b**) Dependence of critical thickness of transition from 2D to 3D growth for Ge wetting layer on Si(001) on growth temperature.

Figure 5 presents the experimental results for the 1/*N* parameter and the critical thickness for the transition from 2D to 3D growth as functions of temperature in the range 200–800 °C. At low temperatures (~200 °C), the short diffusion length of adatoms leads to their occupation of less energetically favorable positions, and growth is predominantly island-like. A relatively small number of dimer vacancies is observed, indicating inefficient strain relaxation and resulting in a reduced critical thickness of the wetting layer (Figure 5).

The increase in the 1/*N* parameter in Figure 5 indicates that raising the growth temperature up to ~550 °C promotes more efficient relaxation of elastic strain within the system, which is associated with a higher concentration of dimer vacancies [40]. The diffusion length of adatoms increases, allowing them to occupy more energetically favorable positions. As a consequence of strain reduction, the critical thickness for the transition from two-dimensional to three-dimensional growth shifts to higher values.

As seen in Figure 5, a decrease in the 1/*N* parameter is observed with further temperature increase. In this regime, the relationship between the 1/*N* parameter and the critical thickness of the wetting layer becomes less clear, as the process becomes nonequilibrium and is governed by several kinetic factors: increased adatom diffusion length along the surface, atomic jumps to overlying layers, the emergence of additional strain relaxation mechanisms, and the formation of *M* × *N* structures followed by their transition to 3D islands.

**Figure 6:**
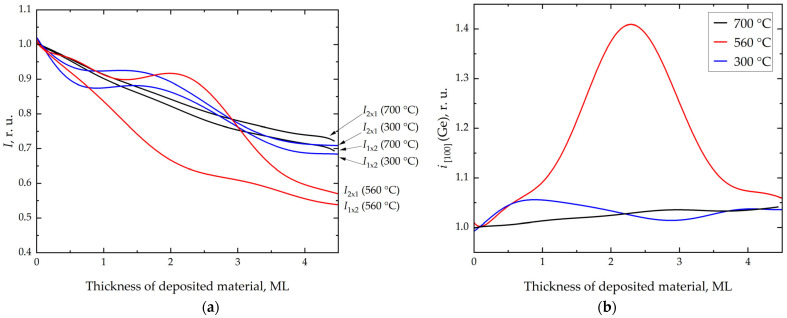
Dependences of (**a**) intensities of reflexes *I*_2×1_ and *I*_1×2_ and (**b**) *I*_2×1_ to *I*_1×2_ ratio (*i*_[100]_) on the thickness of deposited germanium during growth on Si(001) for various temperatures: 300 °C, 560 °C, and 700 °C.

Heteroepitaxial growth of Ge on Si(001) was also studied by RHEED in the [100] azimuth. The time dependences of the intensities *I* of reflections from the 2 × 1 and 1 × 2 superstructures were obtained, and the ratio of these intensities (*i*_[100]_ = *I*_2×1_/*I*_1×2_) was analyzed as a function of germanium coverage. These dependences at various temperatures are shown in Figure 6.

As shown in Figure 6a, *I*_1×2_ decreases monotonically until it vanishes, whereas *I*_2×1_ begins to decrease only after a certain time. The monotonic decrease in *I*_1×2_ indicates that the surface area of type A terraces progressively decreases relative to that of type B terraces. Under ideal conditions—i.e., in the absence of Kikuchi lines, background illumination, and other artifacts, and assuming perfect incorporation of adatoms into the lattice—*I*_2×1_ would be expected to decrease simultaneously with *I*_1×2_. In the present case, however, the surface structure is influenced not only by diffraction-related effects but also by lattice mismatch between Ge and Si. This mismatch leads to the formation of short dimer rows separated by vacancy sites, resulting in a modified surface morphology. A 2 × *N* superstructure forms, followed by the development of an *M* × *N* structure [56]. Thus, the initially smooth surface gradually evolves into a three-dimensional morphology. An increase in *I*_2×1_, associated with the expansion of type B terrace area, in combination with the factors described above, leads to the behavior observed in Figure 6.

**Figure 7:**
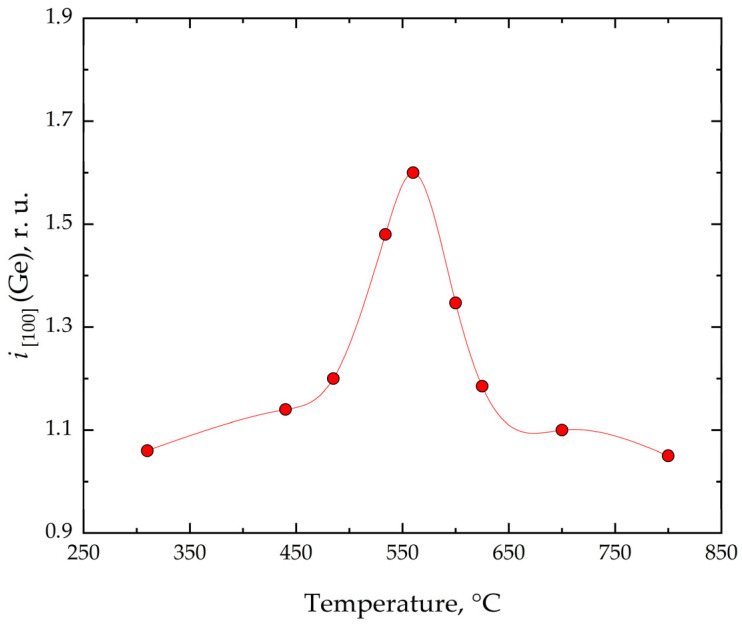
Temperature dependences of *I*_2×1_ to *I*_1×2_ ratio (*i*_[100]_) during heteroepitaxial Ge/Si(001) growth.

The experimental results obtained for Ge deposition on Si(001) at various temperatures are summarized in Figure 7. Comparison with the corresponding data for Si growth (Figure 3) shows that the overall trends are largely similar for both materials. A detailed analysis of the bimodal nature of intensity oscillations during Ge growth was not feasible, since after approximately the fourth Ge monolayer, a transition from two-dimensional to three-dimensional growth occurs, resulting in an insufficient number of oscillations. Nevertheless, the data in Figure 7 provide information on terrace areas. From this, it can be concluded that the mechanisms of Ge and Si terraces formation on Si(001) exhibit similar temperature dependences. The observed differences can be attributed to variations in strain relaxation mechanisms, including the formation of 2 × *N* surface reconstructions, as well as differences in adatom diffusion lengths between Ge and Si.

Thus, the length of the dimer rows in the 2 × *N* superstructure, the onset of their formation, and their evolution during heteroepitaxial growth of Ge on Si(001) are strongly temperature-dependent. The obtained results indicate that achieving optimal surface morphology requires careful selection of the deposition temperature, taking into account both growth kinetics and elastic strain relaxation.

### 3.3. SEM and AFM Analysis of Ge Quantum Dots on Si(001)

**Figure 8:**
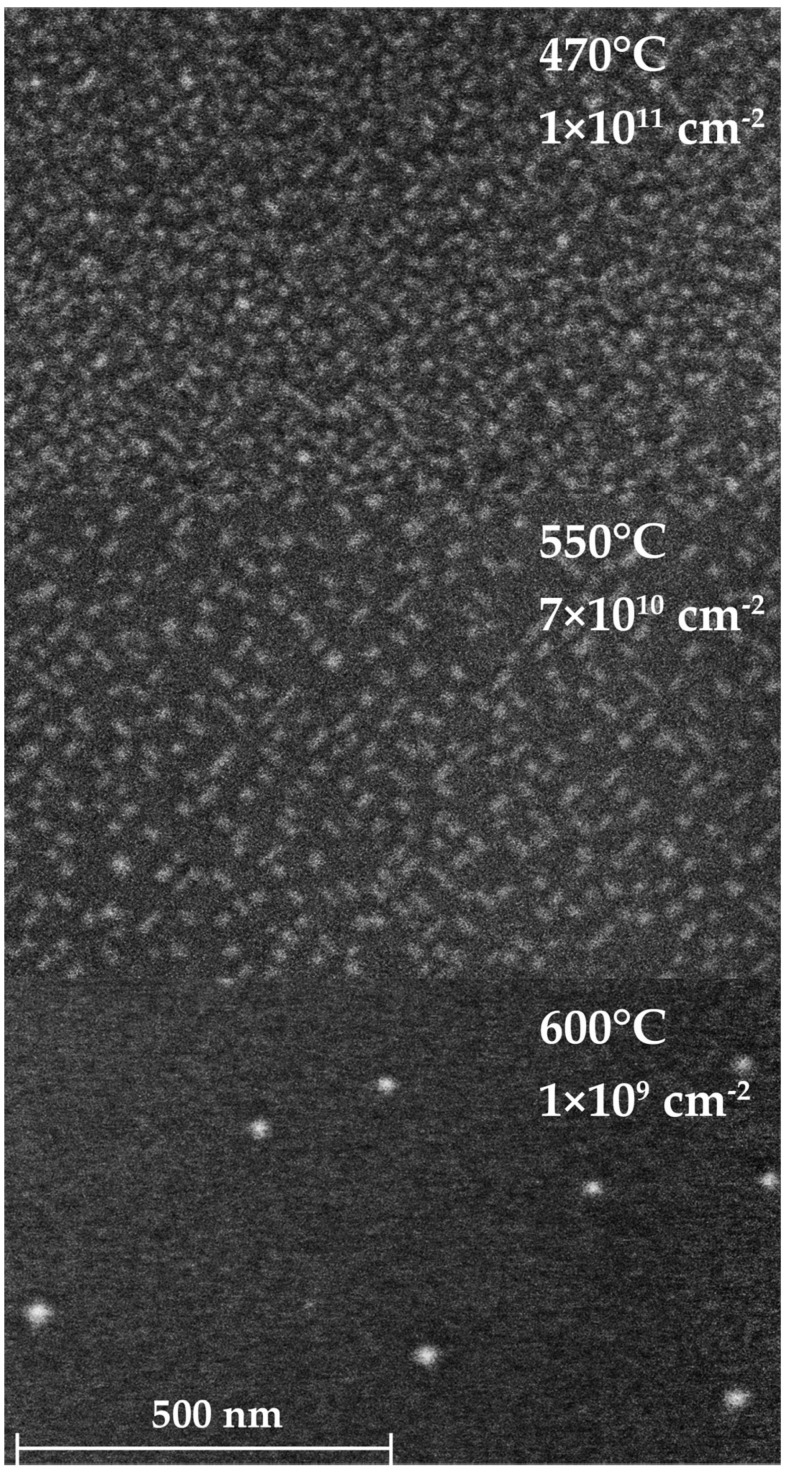
SEM images with values of surface density of germanium islands grown on Si(001) at various temperatures: 470 °C, 550 °C, and 600 °C. The scale bar is the same for all SEM images.

Based on the data presented in Figure 3, Figure 5 and Figure 7, the optimal temperature for the formation of quantum dots is 550 °C, since at this temperature steps of different types are maximally close, while the dimer rows remain relatively short. To compare quantum dot parameters, samples of Ge quantum dots on Si(001) grown at 470 °C (sample 1), 550 °C (sample 2), and 600 °C (sample 3) were examined using scanning electron microscopy (Figure 8) and atomic force microscopy (Figures S3 and S4).

**Figure 9:**
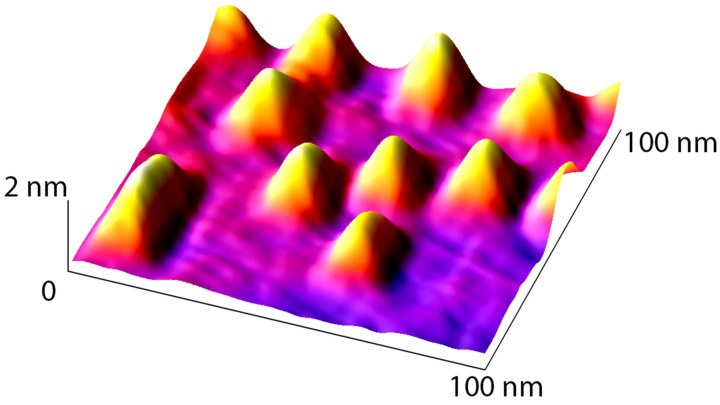
AFM image (3D view) of the sample with Ge islands on Si(001) grown at 550 °C.

During Ge growth on Si(001), hut clusters form via self-organization of atoms following completion of the wetting layer. Hut clusters are characterized by small sizes (1–5 nm in height and 10–50 nm in lateral dimensions) (Figure 9) and pyramidal faceting with a sidewall angle of approximately 11° (Figure S5). The formation of these facets is also confirmed by inclined streaks in the RHEED patterns (Figure 4a). Hut clusters are further classified into two types: wedge-shaped (elongated) and pyramidal [57]. While pyramidal clusters have square bases, wedge-shaped clusters have rectangular bases.

The samples differ in both the density of quantum dot per unit area and the geometric parameters of the nanoclusters. The quantum dot density is on the order of 10^11^ cm^−2^, which is close to the theoretical limit for islands of this size. The density decreases with increasing temperature from 550 °C to 600 °C. The quantum dot densities for samples 1 and 2 are comparable; however, as shown in Figure 8, sample 1 contains a larger number of incompletely formed quantum dots.

**Figure 10:**
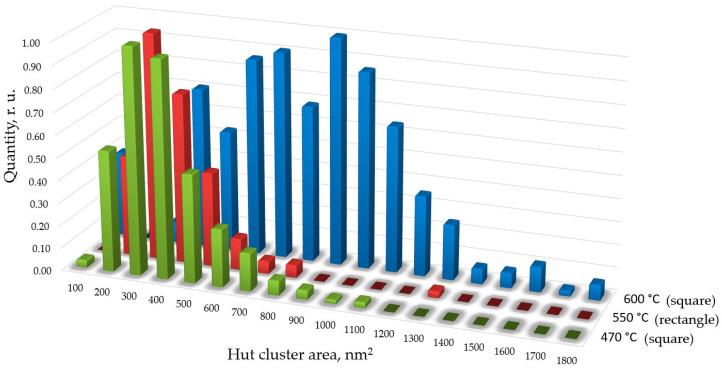
Distribution function of square and rectangular Ge hut clusters on Si(001) by their base area for various growth temperatures: 470 °C, 550 °C, and 600 °C.

To compare cluster sizes across the samples, size distribution functions of islands by base area were constructed (Figure 10). The number of quantum dots used for the analysis ranged from 500 to 2000, depending on the sample. Since the number of dots differs between samples, the distributions were normalized to unity. Figure 10 shows that sample 1 exhibits a broader size distribution in base area than sample 2. Sample 3 is characterized by larger cluster sizes.

As seen in Figure 10, the majority of quantum dots grown at 470 °C have a base area of approximately 350 nm^2^, with the full range spanning from 100 to 1100 nm^2^. For the sample grown at 550 °C, most quantum dots have a base area of approximately 300 nm^2^, and clusters larger than 1000 nm^2^ are not observed. Sample 3 (600 °C) exhibits quantum dots with sizes distributed over a wider range compared to samples 1 and 2, while the density of quantum dots is relatively low (about 10^9^ cm^−2^).

**Figure 11:**
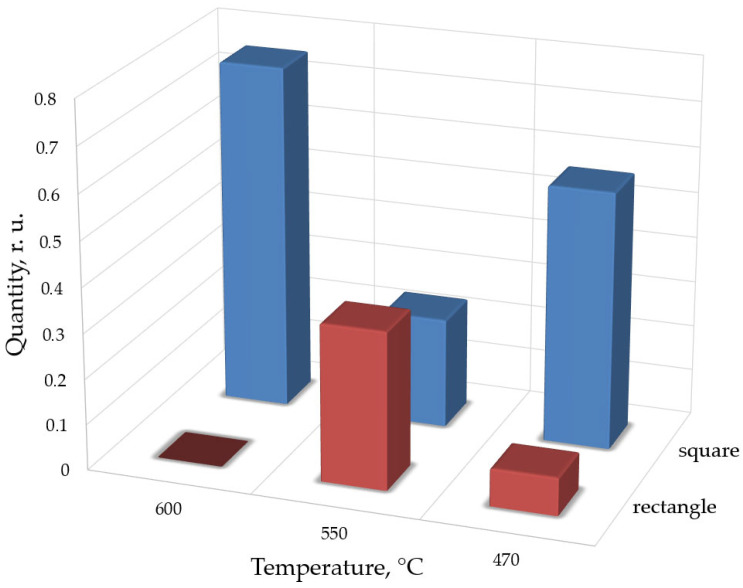
Dependence of relative number of square (blue bars) and rectangular (red bars) islands on growth temperature.

Given the growing interest in quantum wires and elongated nanoclusters [58,59], the distributions of islands by length-to-width ratio were also analyzed. Figure 11 presents the relative fractions of square islands (defined as clusters with the length-to-width ratio less than 1.2) and elongated islands (length-to-width ratio greater than 2). The data indicate that at ~550 °C, hut clusters exhibit greater elongation than at other temperatures. This supports the conclusion that a temperature of approximately 550 °C is optimal for the formation of elongated quantum dots [58].

In summary, the results show that the characteristic size of quantum dots increases with increasing growth temperature. Moreover, quantum dots formed at different temperatures differ significantly in both shape and size distribution. Increasing the growth temperature from 550 °C to 600 °C leads to a substantial decrease in quantum dot density and an increase in their size, while elongation of hut clusters is not observed under these conditions. Conversely, decreasing the temperature from 550 °C to 470 °C results in a slight increase in quantum dot density; however, a significant number of incompletely formed clusters are present. The sample grown at 550 °C exhibits a high density of quantum dots, a large length-to-width ratio of nanoislands, and a relatively narrow size distribution. Therefore, this temperature is particularly favorable for the synthesis of elongated islands and quantum wires.

In the case of Ge/Si QD systems, stability at ambient conditions is generally considered relatively high, primarily due to the covalent bonding and epitaxial integration with the Si substrate. However, this stability is not absolute and depends slightly on surface structure and defect density [60,61]. At the same time, for buried Ge/Si QDs, i.e., structures encapsulated by a Si capping layer, degradation under ambient conditions becomes negligible for most practical purposes. In the presented study, no special growth processes were used to contribute directly to improved durability or resistance to environmental factors of the quantum dots. Experimentally observed broad size distributions caused by probabilistic nature of lateral ordering are directly reflected in the electronic and optical properties of the QD ensemble. In particular, the presence of size and composition fluctuations gives rise to pronounced spectral inhomogeneous broadening and tailing of the density of states, which obscures the discrete energy spectrum expected for ideally monodisperse QDs. This effect is further influenced by intermixing and segregation phenomena during growth and capping, which modify the local composition profile and confinement potential. Strategies to prevent segregation during epitaxy focus on limiting atomic mobility, optimizing kinetic conditions, and managing surface strain. Primary methods include lowering the substrate temperature, increasing the growth rate to trap atoms in place, using surfactant-assisted growth, and employing strain-engineering techniques to reduce the thermodynamic driving force for surface segregation [62,63].

In addition to the density and homogeneity of quantum dots, the optoelectronic characteristics of such nanostructures depend on the size and shape of the quantum dots. These parameters determine the wavelengths of optical radiation that can be absorbed by the detector. Therefore, while monochromatic detectors demand narrow spectra of quantum dots sizes, the use of heterostructures with a broad size distribution of quantum dots will allow the absorption of a wider spectrum of wavelengths, which is very useful for solar energy.

## 4. Conclusions

This study investigates the effect of temperature on the growth characteristics of Si and Ge on Si(001) using reflection high-energy electron diffraction. It is shown that processes occurring during the initial stages of growth determine the subsequent evolution of the heterostructure. A critical temperature of 550 °C is identified, at which the growth mechanisms undergo significant changes. The mechanisms of terrace formation, as well as the emergence and evolution of dimer rows in the 2 × *N* superstructure, are analyzed. Subsequent scanning electron microscopy and atomic force microscopy analysis enabled evaluation of how these processes influence the final morphology and density of the nanoislands.

The experimental results demonstrate a clear relationship between synthesis parameters (in particular, growth temperature), epitaxial growth processes, and nanoisland characteristics. These findings provide a basis for the controlled fabrication of self-assembled quantum dots with desired size and density for subsequent practical applications. Growth of Ge on Si(001) at the critical temperature of 550 °C results in the formation of elongated, high-density quantum dots with a narrow size distribution, which is of significant interest for advanced electronic devices.

## Data Availability

The original contributions presented in this study are included in the article. Further inquiries can be directed to the corresponding author.

## Supplementary Materials

The following supporting information can be downloaded at: https://www.mdpi.com/article/10.3390/nano16090510/s1, Figure S1: AFM image of the surface of Si wafer before pre-epitaxial cleaning; Figure S2: AFM image of the surface of Si wafer after chemical cleaning; Figure S3: AFM image of the sample with Ge islands grown on Si(001) at 470 °C; Figure S4: AFM image of the sample with Ge islands grown on Si(001) at 550 °C; Figure S5: AFM height profiles of typical (a,b) pyramidal and (c,d) elongated clusters along lines of corresponding colors marked in Figure S4.
