# Supplementary material for: Initial and Middle Stages of Quantum Dots Growth: From Dynamics of Superstructures to Island-Size Distributions
Source: Nanomaterials (Basel). 2026 Apr 23;16(9):510. doi: 10.3390/nano16090510 (PMC13165191; doi:10.3390/nano16090510)
Supplement: Supplementary file 1 [file nanomaterials-16-00510-s001.zip › nanomaterials-4221179-supplementary.pdf]

Article

Initial and Middle Stages of Quantum Dots Growth: From Dynamics of Superstructures to Island-Size Distributions

Olzhas Kukenov, Vladimir Dirko, Kirill Lozovoy * and Andrey Kokhanenko

Department of Quantum Electronics and Photonics, Faculty of Radiophysics, National Research Tomsk State University, Lenin Av. 36, 634050 Tomsk, Russia; okukenov@mail.ru (O.K.); vovenmir@gmail.com (V.D.); kokh@mail.tsu.ru (A.K.)

* Correspondence: lozovoymailbox@gmail.com

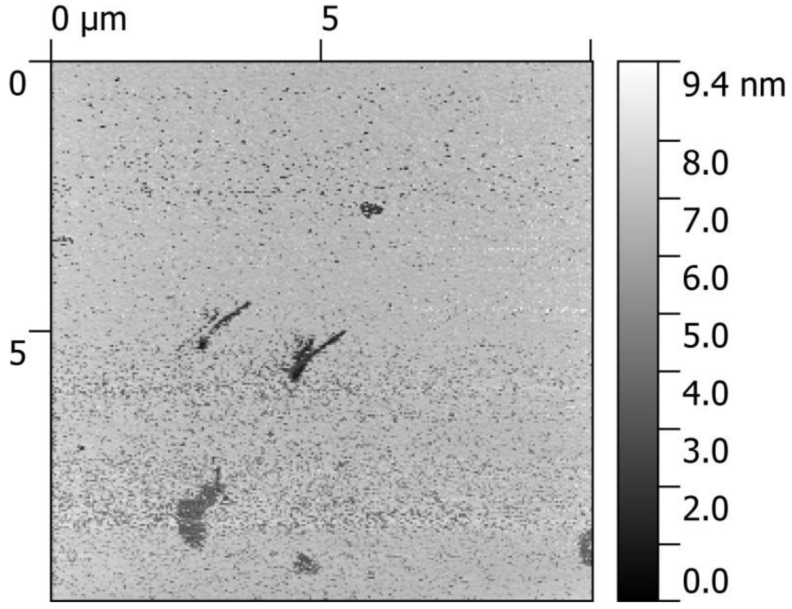

Figure S1. AFM image of the surface of Si wafer before pre-epitaxial cleaning.

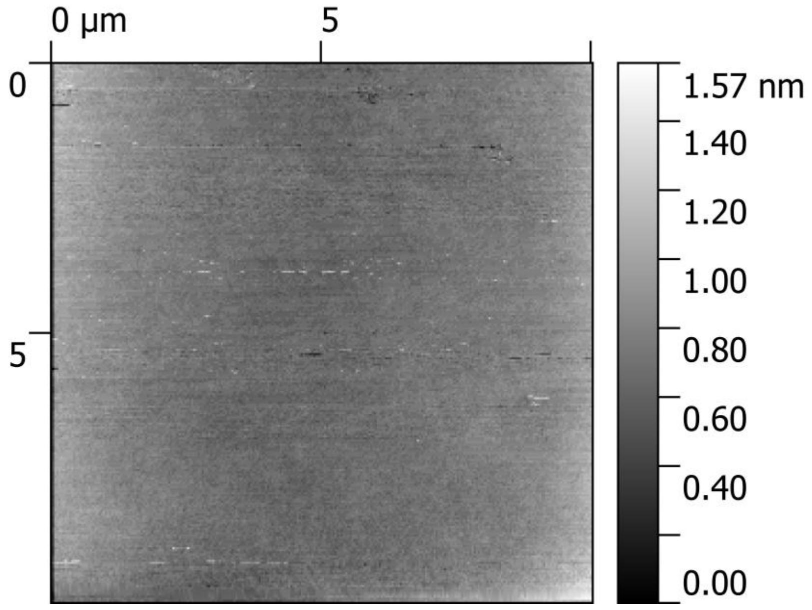

Figure S2. AFM image of the surface of Si wafer after chemical cleaning.

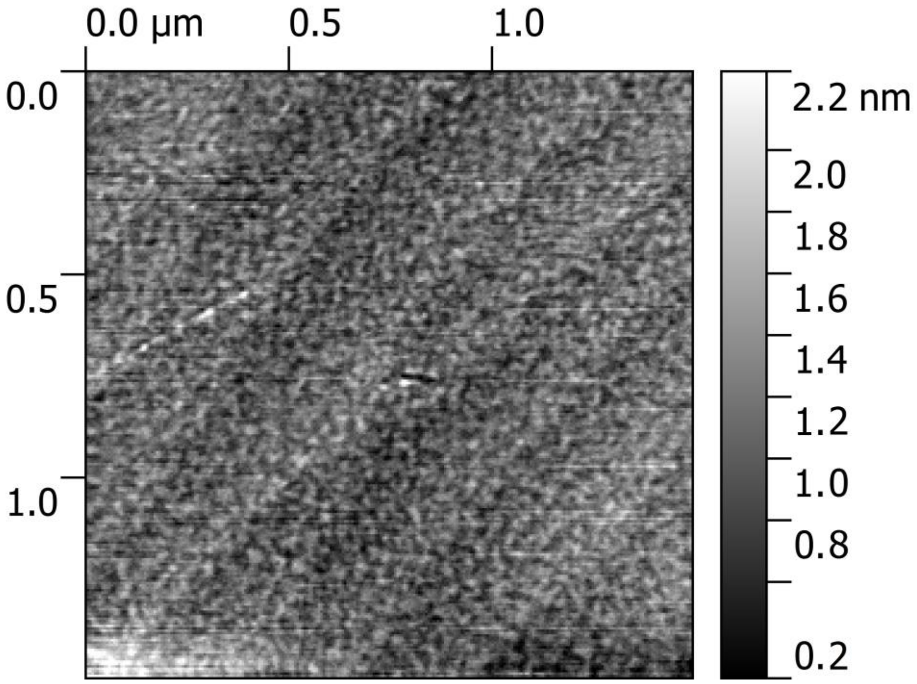

Figure S3. AFM image of the sample with Ge islands grown on Si(001) at 470 °C.

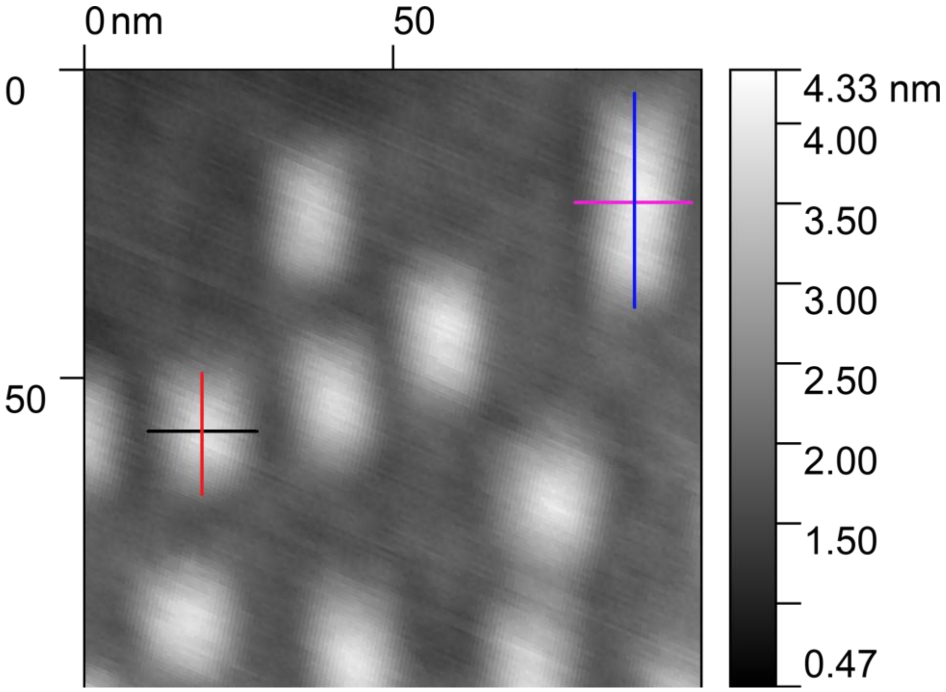

Figure S4. AFM image of the sample with Ge islands grown on Si(001) at 550 °C.

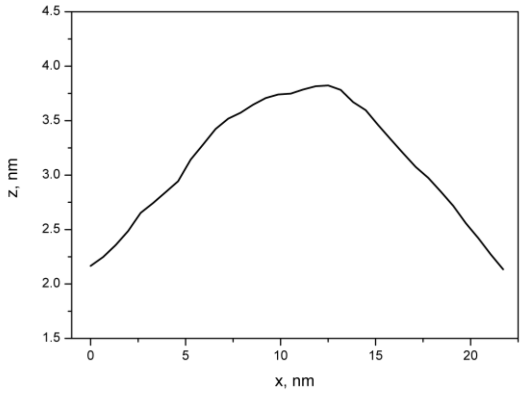

(**a**)

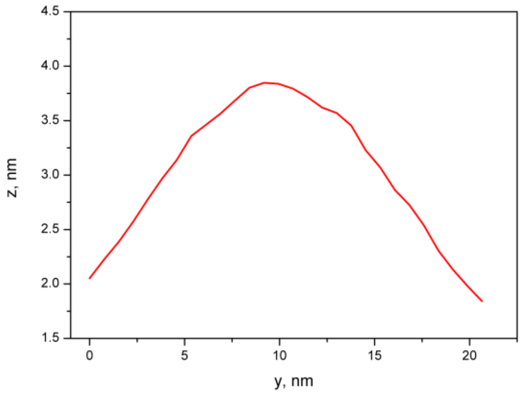

(**b**)

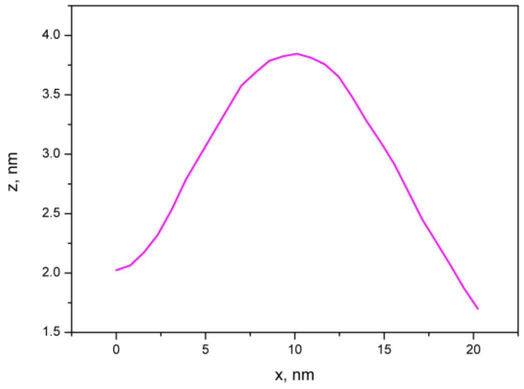

(**c**)

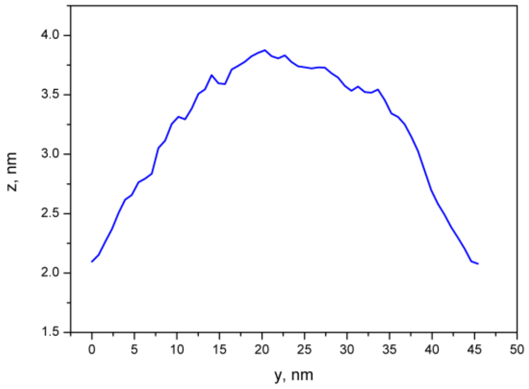

(**d**)

Figure S5. AFM height profiles of typical (**a**), (**b**) pyramidal and (**c**), (**d**) elongated clusters along lines of corresponding colors marked in Figure S4.
